# Epidermal growth factor‐containing fibulin‐like extracellular matrix protein 1 (EFEMP1) suppressed the growth of hepatocellular carcinoma cells by promoting Semaphorin 3B(SEMA3B)

**DOI:** 10.1002/cam4.2144

**Published:** 2019-04-11

**Authors:** Jiangfeng Hu, Bensong Duan, Weiliang Jiang, Sengwang Fu, Hengjun Gao, Lungen Lu

**Affiliations:** ^1^ Department of Gastroenterology Shanghai General Hospital Shanghai Jiaotong University School of Medicine Shanghai China; ^2^ Endoscopy Center Shanghai East Hospital Tongji University School of Medicine Shanghai China; ^3^ Department of Gastroenterology Tongji Hospital Tongji University School of Medicine Shanghai China

**Keywords:** apoptosis, epidermal growth factor‐containing fibulin‐like extracellular matrix protein 1, Hepatocellular carcinoma, proliferation

## Abstract

**Aim:**

Epidermal growth factor‐containing fibulin‐like extracellular matrix protein 1(EFEMP1) has been found to be involved in the occurrence and development of many cancers. The relationship between EFEMP1 and the development of hepatocellular carcinoma (HCC) and the molecular mechanism are not fully understood.

**Methods:**

Real‐time polymerase chain reaction (PCR) and tissue microarray were used to detect the expression of EFEMP1 in HCC cell lines and tissue. Methylation‐specific PCR assay was used to measure the methylation level of EFEMP1 in HCC cell lines and tissue. To study the function of EFEMP1 on cell function, Huh7 and HepG2 were infected with lentiviral particles expressing EFEMP1. MTT assay and colony formation assay were used to examine the effect of EFEMP1 on cell proliferation. Annexin‐VAPC/7‐AAD double were used to detect the effect of EFEMP1 on cell apoptosis. To further detect the effect of EFEMP1 on the development of HCC in vivo, we performed the tumor formation experiment in nude mice. Gene chip was used to detect the expression profile of Huh7 and HepG2 overexpressing EFEMP1. To further screen out the differences, GO analysis and pathway analysis were performed. To study the effects of SEMA3B, specific siRNA was used to inhibit the expression of SEMA3B. Chi‐squared test and rank sum test were used to analyze the relationship between EFEMP1 expression and HCC clinical characteristic.

**Results:**

The study found that the expression of EFEMP1 was significantly decreased in HCC cell lines and HCC tissues. The expression level of EFEMP1 was related to the TNM (the extent of the tumor, the extent of spread to the lymph nodes, the presence of metastasis) stage and the prognosis of patients with HCC. The decrease of protein expression suggested that the patient prognosis was worse, and the protein level of EFEMP1 may be an independent factor in the prognosis of HCC patients. Promoter methylation may be one of the reasons for EFEMP1 inhibition. EFEMP1 could inhibit the proliferation of HCC cells and promoted the apoptosis of HCC cells to regulate the development of HCC. And EFEMP1 promoted the apoptosis of HCC cells mainly through the mitochondrial apoptosis pathway. EFEMP1 may inhibit the proliferation of HCC cells through the SEMA3B gene in the Axon guidance pathway.

**Conclusion:**

In summary, our research revealed the regulation of EFEMP1 on cell proliferation and apoptosis in HCC. EFEMP1 may suppress the growth of HCC cells by promoting SEMA3B.

## 1 INTRODUCTION

Primary liver cancer is one of the most common malignancies in China. Early symptoms are occult, and many patients have been found to have advanced stages of liver cancer. Its morbidity, mortality, and death toll have long been among the top three types of malignant tumors.^1^, ^2^, ^3^, ^4^ Hepatocellular carcinoma (HCC) accounts for about 85%‐90% of primary liver cancer. The etiology and pathogenesis of primary liver cancer are still not fully understood, and most viewpoints are considered to be multi‐factor complex and multistep complex diseases.^5^

Epidermal growth factor‐containing fibulin‐like extracellular matrix protein 1 (EFEMP1), also known as Fibulin‐3, is a member of the Fibulin glycoprotein family. EFEMP1 was first found in aged fibroblasts. The main role of EFEMP1 is to maintain the stability of the basement membrane and the integrity of the extracellular matrix. Existing studies have shown that EFEMP1 plays an important role in the progression of multiple tumors.^6^ In cervical cancer, high expression of EFEMP1 promoted the proliferation, invasion, and adhesion of tumor cells.^7^ In pancreatic cancer, EFEMP1 activated the mitogen‐activated protein kinase (MAPK) and protein Kinase B (PKB/AKT) pathways by binding to the EGF receptor to promote tumor proliferation.^8^ However, in some other tumors, EFEMP1 was considered to play a role in tumor suppression. For example, in lung cancer, EFEMP1 inhibited tumor invasion by inhibiting MMP‐7 expression.^9^ In nasopharyngeal carcinoma, EFEMP1 inhibited cell migration and invasion by affecting the activity of AKT.^10^ In addition, EFEMP1 could be detected abnormally in the serum of tumor patients,^11^, ^12^ so it may serve as an important marker for clinical diagnosis of cancer. These studies suggested that EFEMP1 may be a molecular detection marker and an important drug target for early diagnosis of tumors. However, whether it is related to the tumorigenesis or development of HCC and the molecular mechanism of action is not completely clear. It has been reported that in the process of HCC, methylation of the promoter region causes a decrease in the expression of EFEMP1.^13^ There were several shortcomings in this study. Firstly, the sample size was too small. It was unknown whether EFEMP1 was really related to HCC. Secondly, the relationship between expression quantity and clinical pathological characteristics had not been directly studied. Thirdly, there was no relevant research on cell function.

In this study, we examined the expression and significance of EFEMP1 in tumor tissues of HCC patients, analyzed the effects of EFEMP1 on the biological behavior of HCC cells, and initially studied the mechanism of EFEMP1 on the development of HCC. The initial search for the regulatory mechanism of EFEMP1 on HCC provides a theoretical basis for the possible future clinical applications.

## 2 METHODS

### 2.1 Cell culture and plasmid

Cell lines were acquired from Cell Bank at Shanghai Institutes for Biological Sciences of Chinese Academy of Sciences. Cells were cultured in Dulbecco's modified Eagle's medium (DMEM, Thermo, Waltham, MA, USA) supplemented with 10% fetal bovine serum (FBS, Gibco, Grand Island, NY, USA). Expression plasmid for EFEMP1 was kindly gifted from Yuanjie Hu.^14^, ^15^, ^16^

### 2.2 Tissue microarray

Tissue microarray was bought from the Biobank of National Engineering Center for Biochip at Shanghai. To measure the staining intensity of tissue, the semiquantitative method was used. The staining intensity (I) was divided into four grades: 0, none; 1, poor; 2, moderate, and 3 strong. The percentage of positive cells (P) was scored as: 0, 0%; 1, 1%‐25%; 2, 26%‐50%; 3, 50%‐75%; and 4, > 75%. The total score (I + P) ≤ 4 was defined as low expression, and score >4 was defined as high expression. This study was approved by the Ethics Committees of National Engineering Center for Biochip in Shanghai.

### 2.3 RT‐PCR for mRNA expression

Total RNA was extracted from tissues and cells using Trizol as described previously.^17^, ^18^ To detect the expression of mRNA, 500 ng of total RNA was used to synthesize cDNA by reverse transcription using PrimeScript RT Master Mix. Primer sequences were listed as follows: GAPDH (forward: 5‐AGAAGGCTGG GGCTCATTTG‐3; reverse: 5‐AGGGGCCATCCACAGTCTTC‐3); EFEMP1 (forward: 5‐GCAGGCTACGAGCAAAGTGAAC‐3, reverse: 5‐GCTTCTGATAT CCAGGAGGGCA‐3); Ki‐67 (forward: 5‐CCTGCTCGACCCTACAGAGTG‐3, reverse: 5‐GTTGCTCCTTCACTGGGGTC‐3); SEMA3B (forward: 5‐CTTCGGCT CTCCTTTCAAGA‐3, reverse: 5‐CAAGGCTTCATAACAGCAGGT‐3)

### 2.4 MTT assay

Cells were seeded in the 96‐well plate at a density of 3000/well. After 12, 24, 48, 72 hours, 100 μL MTT was added. Then cells were incubated for 4 hours. Hundred microliters of DMSO was added into each well. After placed on a shaker at low speed for 15 minutes, the microplate reader was used to detect the absorbance at 490 nm.

### 2.5 Colony formation assay

The method of colony formation experiment referred to the previous research.^19^ Cells were seeded into a 3.5 cm dishes at a density of 2000 per dish and cultured in an incubator. When macroscopic clones appeared in the culture dish, culture dish was removed and washed once with PBS. Methanol was added for 30 minutes to fix cells. Crystal violet staining solution was added for 20 minutes, and then the staining solution was slowly washed with running water and air‐dried. The number of cells was counted by the naked eye and divided by the number of cells laid to obtain the colony fomration rate.

### 2.6 Flow cytometry

Cells were collected into a 10 mL centrifuge tube at a density of 1‐5 × 10^6^/mL for each group and washed twice with PBS. Then cells were resuspended with labeling solution and incubated in the dark for 10‐15 minutes at room temperature. After centrifuging, fluorescent dye was added for 20 minutes at 4°C. Flow cytometric analysis was used to quantify cell apoptosis.

### 2.7 Tunel staining

Cells were fixed in 4% paraformaldehyde overnight. To improve cell permeability, 1% Triton‐100 was added for 15 minutes at room temperature. After fixed with 3% H_2_O_2_‐methanol solution for 15 minutes, cells were incubated with TdT enzyme reaction solution in the dark for 1 hour at 37°C. Streptavidin‐TRITC was added in the dark for 30 minutes at 37°C. Hoechst 33 258 was used to stain. The number of apoptotic cells in five randomly chosen microscopic fields was counted.

### 2.8 Western blotting

Cells were lysed in SDS sample buffer. Protein concentration was determined using a bicinchoninic acid method. Proteins were separated using SDS‐PAGE, transferred to the pure nitrocellulose blotting membranes, and detected with appropriate antibodies as described below. Antibodies used in the experiment were: anti‐GAPDH (Sigma), anti‐EFEMP1 (Abgent),anti‐Bad (Biogot),anti‐Bid (Biogot), anti‐Bcl‐2 (Biogot), anti‐Caspase‐3 (Biogot), anti‐Caspase‐8 (Biogot), and anti‐Caspase‐9 (Biogot).

### 2.9 Nude mouse tumor formation experiment

Male nude mice of 4 weeks old were purchased from Shanghai Slaccas. The cells were resuspended in a mixture of 1 × PBS and matrigel with a volume ratio of PBS to matrigel of 9:1. The injection volume of each mouse was 0.2 mL per needle, and each injection of 1 × 10^7^ cells was subcutaneously injected into the neck. Nude mice injected with HepG2 cells were terminated at 4 weeks, while nude mice injected with Huh7 cells were terminated at 5 weeks. The tumor mass was taken out and weighed.

### 2.10 β‐galactosidase staining experiment

Cells were fixed with galactosidase staining fixative for 30 minutes at room temperature and stained with staining working solution overnight at 37°C incubator. On the next day, the number of cytoplasmic blue‐stained cells was counted under a light microscope, and four randomly fields were taken from each group. The positive rate of cell staining was calculated.

### 2.11 Statistical analysis

The experimental data were analyzed and plotted using SPSS 20.0 and GraphPad Prism 5.0 software. Data results were expressed as mean ± standard deviation, using paired *t* test, unpaired *t* test, chi‐squared test, Wilcoxon signed rank test, and Pearson's correlation analysis, *P *<* *0.05 was considered statistically significant.

## 3 RESULTS

### 3.1 mRNA level of EFEMP1 in HCC tissues and HCC cells

**Figure 1:**
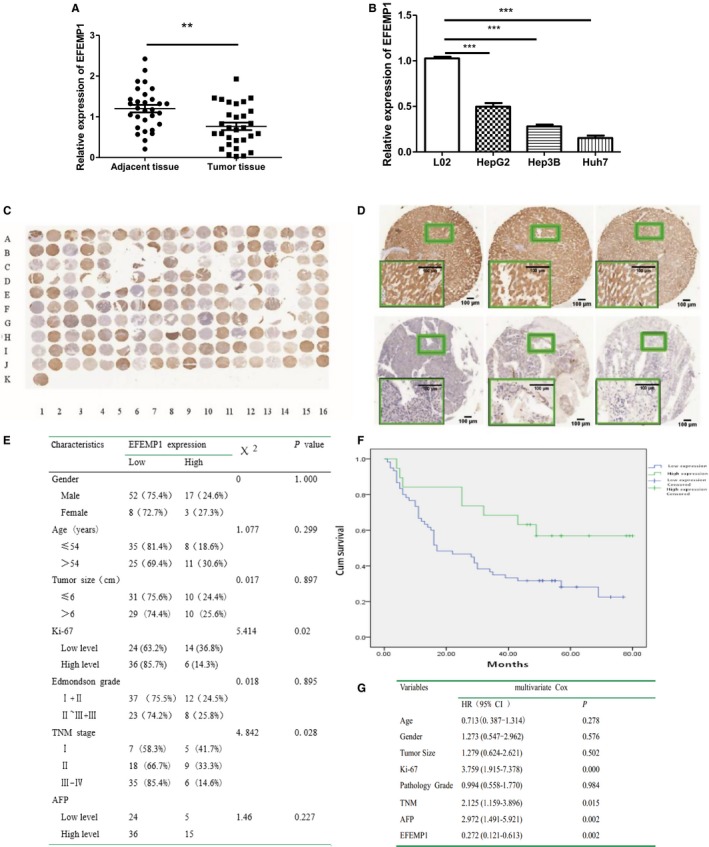
A, mRNA expression of EFEMP1 in liver cancer tissues and adjacent noncancerous tissues. B, mRNA expression of EFEMP1 in normal liver cell and liver cancer cell lines. C and D, HCC tissue chip, odd‐numbered represented liver cancer tissues, and even‐numbered represented adjacent noncancerous tissues. EFEMP1 staining of tissue microarray, the results showed that the staining intensity and positive rate of EFEMP1 protein in liver cancer tissues were significantly lower than those in adjacent noncancerous tissues. E, Relationship between the protein expression level of EFEMP1 and clinical pathology information. The results showed that EFEMP1 protein level was associated with Ki‐67 and TNM stage, regardless of age, gender, tumor size, grade, and AFP level. F, Kaplan‐Meier survival analysis suggested a positive correlation between EFEMP1 expression and survival time in HCC patients. Low expression of EFEMP1 suggested a worse prognosis in patients. G, Multivariate Cox regression model analysis suggested that TNM stage, protein levels of Ki‐67, AFP and EFEMP1 may be independent prognostic factors for HCC patients. The protein level of EFEMP1 was a protective factor for HCC (*P *=* *0.002). *P < 0.05, **P  <  0.01, ***P  <  0.001

We used real‐time polymerase chain reaction (RT‐PCR) to detect the mRNA level of EFEMP1 in 30 pairs of human liver cancer tissues and adjacent noncancerous tissues. The results showed that there was lower mRNA level of EFEMP1 in 18(60%) of the cancer tissues compared with adjacent noncancerous tissues, and the difference was statistically significant (Figure 1A). Subsequently, we detected the expression of EFEMP1 in normal liver cell L02 and liver cancer cell lines HepG2 and Huh7. The results showed that the mRNA level of EFEMP1 in the two HCC cell lines was lower than that in the normal liver cell L02 (Figure 1B).

### 3.2 Protein level of EFEMP1 in HCC tissues

The results of the previous experiments suggested that the mRNA level of EFEMP1 was significantly downregulated during hepatocarcinogenesis. To further validate our inference and study the relevance of EFEMP1 and clinical pathology, the sample size was expanded. The HLiv‐HCC180Sur‐02 chip contained 90 pairs of HCC tissues (odd‐numbered represented HCC tissues (eg,: A1, B1… J1, A3…), and even‐numbered (eg,: A2, B2…J2, A4…) represented the corresponding adjacent noncancerous tissues). The results of the tissue microarray showed that the staining intensity and positive rate of EFEMP1 protein in HCC tissues were significantly lower than those in adjacent noncancerous tissues (Figure 1C,D).

### 3.3 Correlation between the protein expression level of EFEMP1 and clinical features of HCC patients

Judging criteria for tissue chip staining results: comprehensive judgment based on coloring intensity and number of positive cells. Among the 90 cases of HCC, the expression of EFEMP1 was low in 60 cases (67.8%), and high in 20 cases (21.1%), six cases were detached, and clinical data were incomplete in four cases. Chi‐squared test and rank sum test were used to analyze the correlation between EFEMP1 protein level and various clinicopathological parameters such as age, sex, tumor size, and TNM stage of HCC patients. The results showed that the expression level of EFEMP1 in HCC was significantly correlated with Ki‐67 protein level (*P *=* *0.02) and TNM stage (*P *=* *0.028) (Figure 1E). Among the 80 cases of HCC, the number of death was 52, accounting for 65%. The median overall survival of the patients was 29 months. The median survival of patients with low expression of EFEMP1 was 17 months, while the median survival of patients with high expression of EFEMP1 protein was 48 months. Kaplan‐Meier survival analysis suggested a positive correlation between EFEMP1 expression and survival time in HCC patients (Figure 1F). Low expression of EFEMP1 suggested a worse prognosis in patients (Logrank test, *P *<* *0.05). Multivariate Cox regression model analysis suggested that TNM stage, protein levels of Ki‐67, AFP and EFEMP1 may be independent prognostic factors for HCC patients. The protein level of EFEMP1 was a protective factor for HCC (*P *=* *0.002) (Figure 1G).

### 3.4 Detection of DNA methylation in the EFEMP1 promoter region in liver cancer cells and tissues

**Figure 2:**
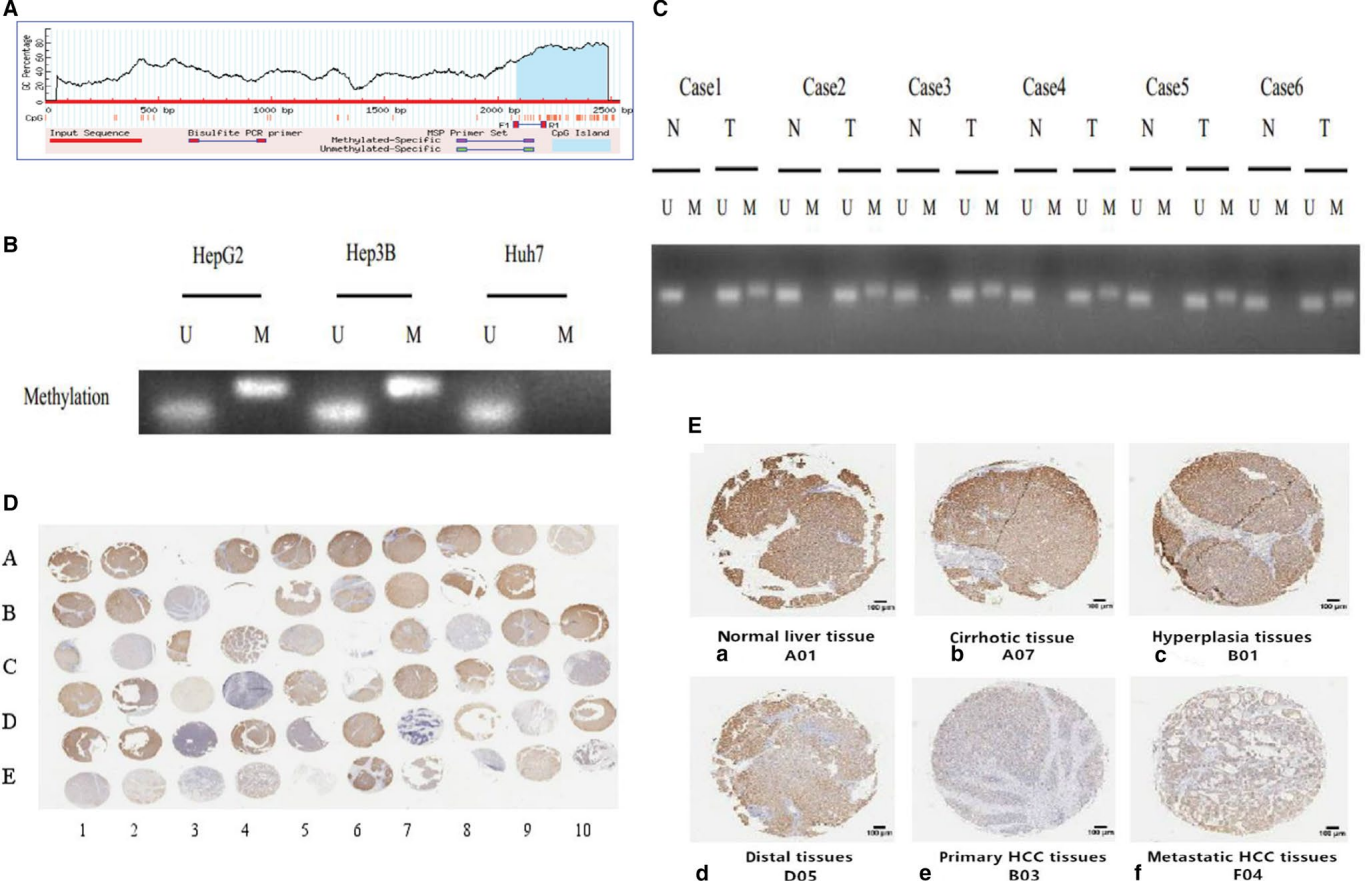
A, The biological software predicts that CpG islands were enriched in the first exon region of EFEMP1 promoter. B, Methylation status of EFEMP1 promoter region in HepG2 and Huh7 cells. C, Methylation status of EFEMP1 promoter region in HCC tissue. D and E, Immunohistochemistry result of EFEMP1 protein in liver tissue. (a) Normal liver tissue. (b) Cirrhotic tissue. (c) Liver hyperplasia tissue. (d) Liver distal tissues. (e) Primary HCC tissues. (f) Metastatic HCC tissues

The previous results suggested that the expression of EFEMP1 was inhibited in liver cancer. In other tumors, it has been reported that EFEMP1 transcription is inhibited due to DNA methylation. We hypothesized that the inhibition of EFEMP1 expression in HCC may also be associated with DNA methylation. To further investigate the location of DNA methylation, we first analyzed the EFEMP1 promoter region by biological software and found that CpG islands were enriched in the first exon region (Figure 2A). The corresponding primers were designed in this region, and the methylation of EFEMP1 in the promoter regions of HepG2 and Huh7 cell lines was detected by MSP. The results showed that in HepG2 cells, the EFEMP1 promoter region showed mixed methylation status (M + U), and there was no methylation status (U) in Huh7 cells (Figure 2B). The results suggested that low expression of EFEMP1 in HCC may be associated with DNA methylation. Further, we detect the methylation status of EFEMP 1 promoter in HCC tissues. We found that six cases of 10 tumor tissues showed methylation status (Figure 2C).

### 3.5 Protein level of EFEMP1 in liver tissue

The previous results suggested that the mRNA level and protein level of EFEMP1 were significantly inhibited in HCC tissues. Then we used tissue microarrays to detect protein level of EFEMP1 in. The tissue in the chip included two normal liver tissues, seven cirrhotic tissues, two liver hyperplasia tissues, 19 primary HCC tissues, 19 corresponding adjacent noncancerous tissues, four liver distal tissues, and seven metastatic liver cancer tissues. It could roughly reflect the expression changes of EFEMP1 protein during the development of liver disease. The results showed that the staining intensity and positive rate of EFEMP1 in 2 normal liver tissues were the highest, indicating that the level of EFEMP1 protein in normal liver tissues was higher than that in the diseased liver tissues. In addition, we could also see that as the degree of liver lesions worsened, the staining intensity and positive rate of EFEMP1 gradually decreased. In HCC tissues, the expression of EFEMP1 protein was lowest and not even detected. In conclusion, the chip results suggest that EFEMP1 was involved in the process of liver disease, and its expression was significantly reduced during hepatocarcinogenesis (Figure 2D,E).

### 3.6 Effect of EFEMP1 on proliferation of HCC

**Figure 3:**
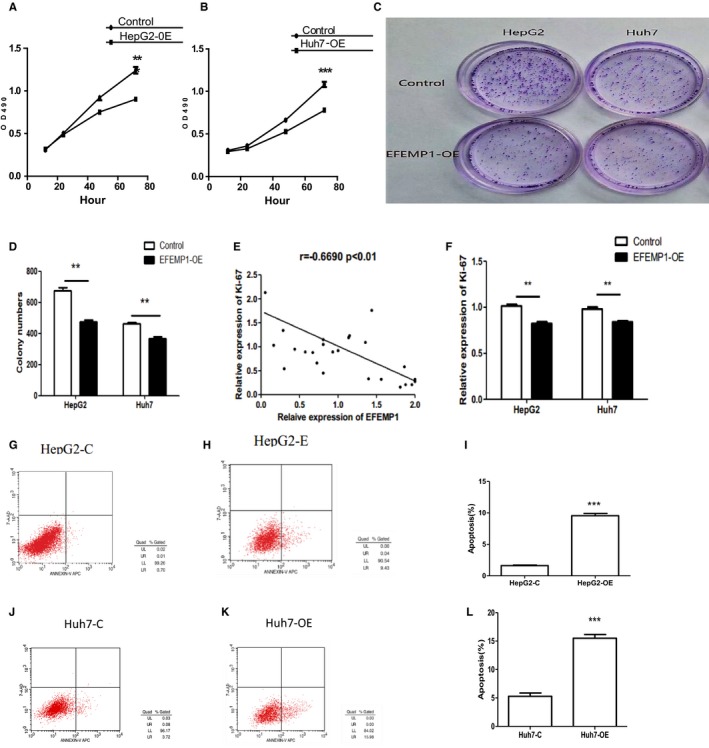
A, MTT assay detected the effect of EFEMP1 on the proliferation of liver cancer cells. When EFEMP1 was overexpressed, the proliferation of HepG2 and Huh7 cells was inhibited. B and C, MTT assay detected the effect of EFEMP1 on the proliferation of liver cancer cells. When EFEMP1 was overexpressed, the proliferation of HepG2 and Huh7 cells was inhibited. D, The correlation analysis showed that the expression of EFEMP1 was negatively correlated with the expression of Ki‐67. E and L, Overexpression of EFEMP1 inhibited the expression of Ki‐67 in liver cancer cells. F‐K, Annexin‐VAPC/7‐AAD double staining assay showed the change of apoptosis rate of liver cancer cells after overexpression of EFEMP1. The results reflected that EFEMP1 could promote the apoptosis of liver cancer cells. *P < 0.05, **P  <  0.01, ***P  <  0.001

The basic biological feature of malignant tumors is the uncontrolled proliferation of tumor cells. There was no previous report on the relationship between EFEMP1 and cell proliferation, but our experimental results showed that the expression of EFEMP1 in HCC tissues was related to the expression of Ki‐67. Ki‐67 is a proliferation‐related gene, suggesting that EFEMP1 may be involved in tumor cell proliferation.^20^ Therefore, cell experiments were subsequently carried out to study the effect of EFEMP1 on the proliferation function of HCC cells. Cells (control group, overexpressing EFEMP1 group) were seeded into 96‐well plates at a density of 3000 cells per well. After 12 h, 24 h, 48 h, and 72 h, MTT reagents were added. Then plates were detected by microplate reader. Based on the measured absorbance, the number of cells was judged to reflect the ability of cell proliferation. The results showed that after overexpressing EFEMP1 in HepG2 and Huh7 cells, the proliferation rate of cells was slower than that of the control cells (Figure 3A,B).

After passage, not every cell could proliferate and form clones. The cells forming clones must be adherent cells with strong proliferative viability. Clonal formation experiments could well reflect cell population dependence and proliferation ability. Therefore, to further verify the effect of EFEMP1 on the proliferation of liver cancer cells as reflected in the MTT assay results, cell clonal formation experiment was performed. HCC cells were inoculated into 3.5 cm cell culture dishes at a density of 1.0x10^3^ cells per dish and incubated in the incubator for 2 weeks. The results showed that the cell clonal formation rate of the EFEMP1 overexpression group was significantly lower than that of the control group (Figure 3C,D). The regulation of EFEMP1 on the proliferation function of HCC cells was further explained.

Analysis of clinical data found that EFEMP1 was not associated with tumor size, but was associated with Ki‐67. Ki‐67 is an antigen associated with cell proliferation and is closely related to mitosis of cells. It is often used as an antigen for labeling cell proliferation. Ki‐67 is expressed in G1, S, G2, and M of cell proliferation and not expressed in G0 phase. Previous tissue microarray results showed that the protein expression level of EFEMP1 was significantly correlated with Ki‐67 protein level. The mRNA level of Ki‐67 in HCC tissues was detected by RT‐PCR. The results reflected that the expressions of Ki‐67 and EFEMP1 showed a linear trend, and there may be a linear relationship (Figure 3E). Subsequently, we detected the expression of Ki‐67 in HCC cells and found that the expression of Ki‐67 in EFEMP1 overexpressing HCC cells was lower than that in the control group (Figure 3F).

### 3.7 Overexpression of EFEMP1 induced apoptosis of HCC cells

**Figure 4:**
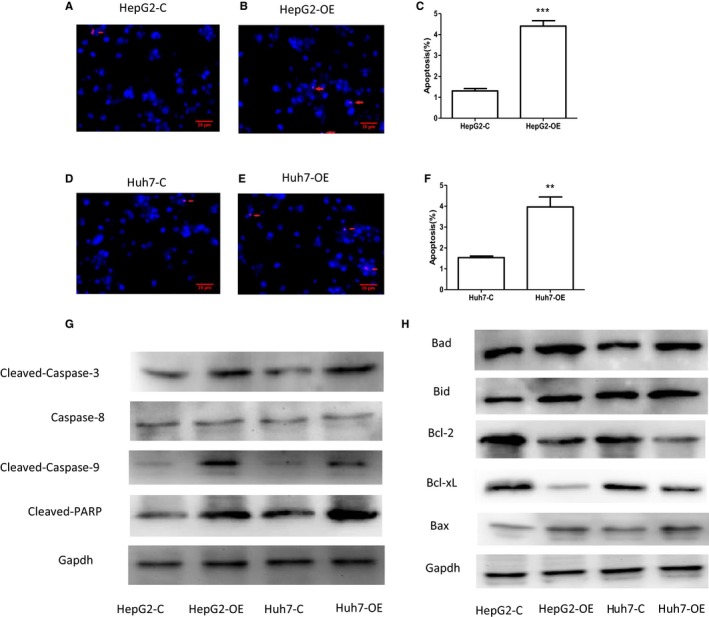
A‐F, Tunel assay for the effect of EFEMP1 on apoptosis of liver cancer cells. The results showed that the proportion of apoptotic cells increased after overexpression of EFEMP1. The arrow refers to apoptotic cells. G, The changes in protein expression of apoptosis‐associated Caspase family members. The results showed that after overexpression of EFEMP1, Caspase‐3 and Caspase‐9 were mainly affected. H, The expression of apoptosis‐related proteins. The results showed that EFEMP1 promoted the expression of proapoptotic proteins and inhibited the expression of anti‐apoptotic proteins. *P < 0.05, **P  <  0.01, ***P  <  0.001

The flow cytometry and tunel assay were performed to detect the effect of EFEMP1 on the apoptosis of HCC cells. Both flow cytometry and tunel assay suggested that the overexpression of EFEMP1 promoted the apoptosis in HCC cells. The results of double staining showed that the apoptosis rate of HepG2 cells and Huh7 cells increased significantly after EFEMP1 overexpressed. The apoptotic rate of HepG2 cell increased from about 0.7% to about 9.5%. The apoptotic rate of Huh7 cell increased from about 3.7% to about 16% (Figure 3G‐L). The effect of EFEMP1 on apoptosis of HCC cells was further examined by Tunel method. Four fields of view for each group were selected for statistical calculations, and the average was obtained. The results showed that EFEMP1 could significantly promote the apoptosis of HCC cells (Figure 4A‐F). To preliminarily study the molecular mechanism by which EFEMP1 regulates apoptosis, we examined the expression of Caspase family members that played an important role in apoptosis. The expression levels of Caspase‐3, Caspase‐8, Caspase‐9, and PARP in cancer cells overexpressing EFEMP1 were detected by Western blotting. The results showed that the increase in degradation products of PARP after overexpression of EFEMP1 indicated the occurrence of apoptosis in HCC cells. Caspase‐3, Caspase‐9 zymogens content decreased, and activated Caspase‐3 and Caspase‐9 increased. The change in Caspase‐8 was not obvious. The role of EFEMP1 in inducing apoptosis of HCC cells was related to the Caspase family and may induce apoptosis of HCC cells through the mitochondrial apoptotic pathway rather than the death receptor pathway (Figure 4G). When cells were affected by internal apoptotic stimulators, such as oncogene activation, DNA damage, etc., they may activate the mitochondrial apoptotic pathway inside the cell and induce apoptosis. In the mitochondrial apoptotic pathway, the permeabilization of mitochondrial outer membrane caused a release of cytochrome C to the cytoplasm, which was mainly regulated by Bcl‐2 family proteins. To investigate whether EFEMP1 has an effect on Bcl‐2 family‐related genes, the expression of Bad, Bid, Bax, Bcl‐2, and Bcl‐xL in HCC cells were detected. The expressions of the proapoptotic genes Bad, Bid, and Bax were increased, while the expressions of the genes for inhibiting apoptosis, Bcl‐2 and Bcl‐xL, were decreased (Figure 4H.).

### 3.8 The effect of EFEMP1 on the growth of HCC cells in vivo test

**Figure 5:**
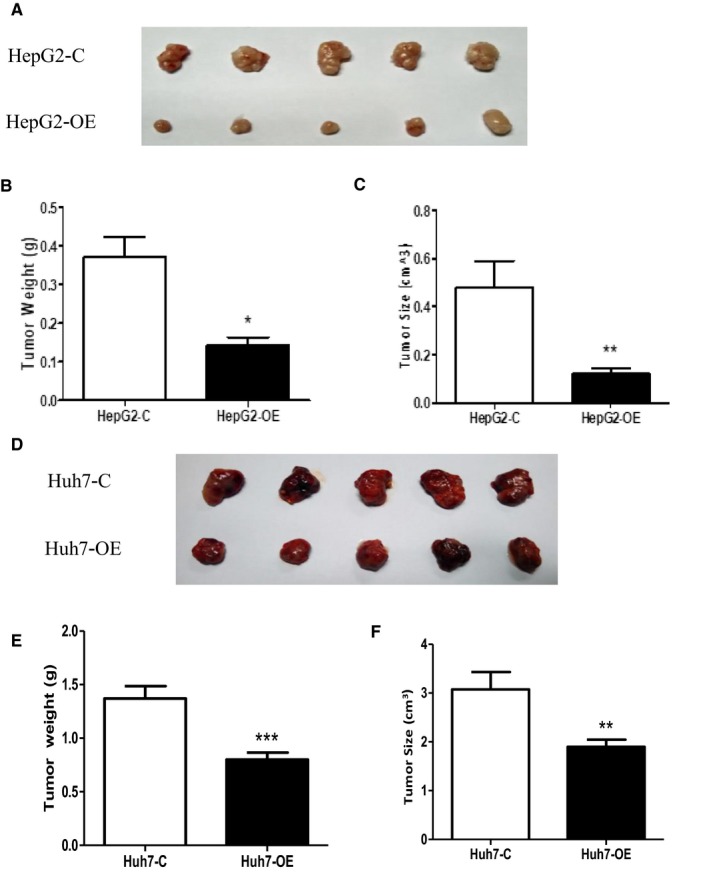
A‐C, HepG2 cells overexpressing EFEMP1 formed smaller tumor volumes and weights than the control group. D‐F, Huh7 cells overexpressing EFEMP1 formed smaller tumor volumes and weights than the control group. *P < 0.05, **P  <  0.01, ***P  <  0.001

To further investigate the effect of EFEMP1 on the growth of HCC cells in vivo, we injected the HCC cell lines overexpressing EFEMP1 into the neck of nude mice. The results suggested that HCC cells overexpressing EFEMP1 grew slower than that of the control group, and the size of the tumor was smaller. Nude mice injected with HepG2 cells were terminated at 4 weeks. Nude mice injected with Huh7 cells were terminated at 5 weeks. After weighing the tumor, it was observed that the tumor size overexpressing the EFEMP1 group was smaller than that in the control group and the tumor weight was also lighter (Figure 5A‐F).

### 3.9 EFEMP1 downstream‐related signaling pathways and genes

**Figure 6:**
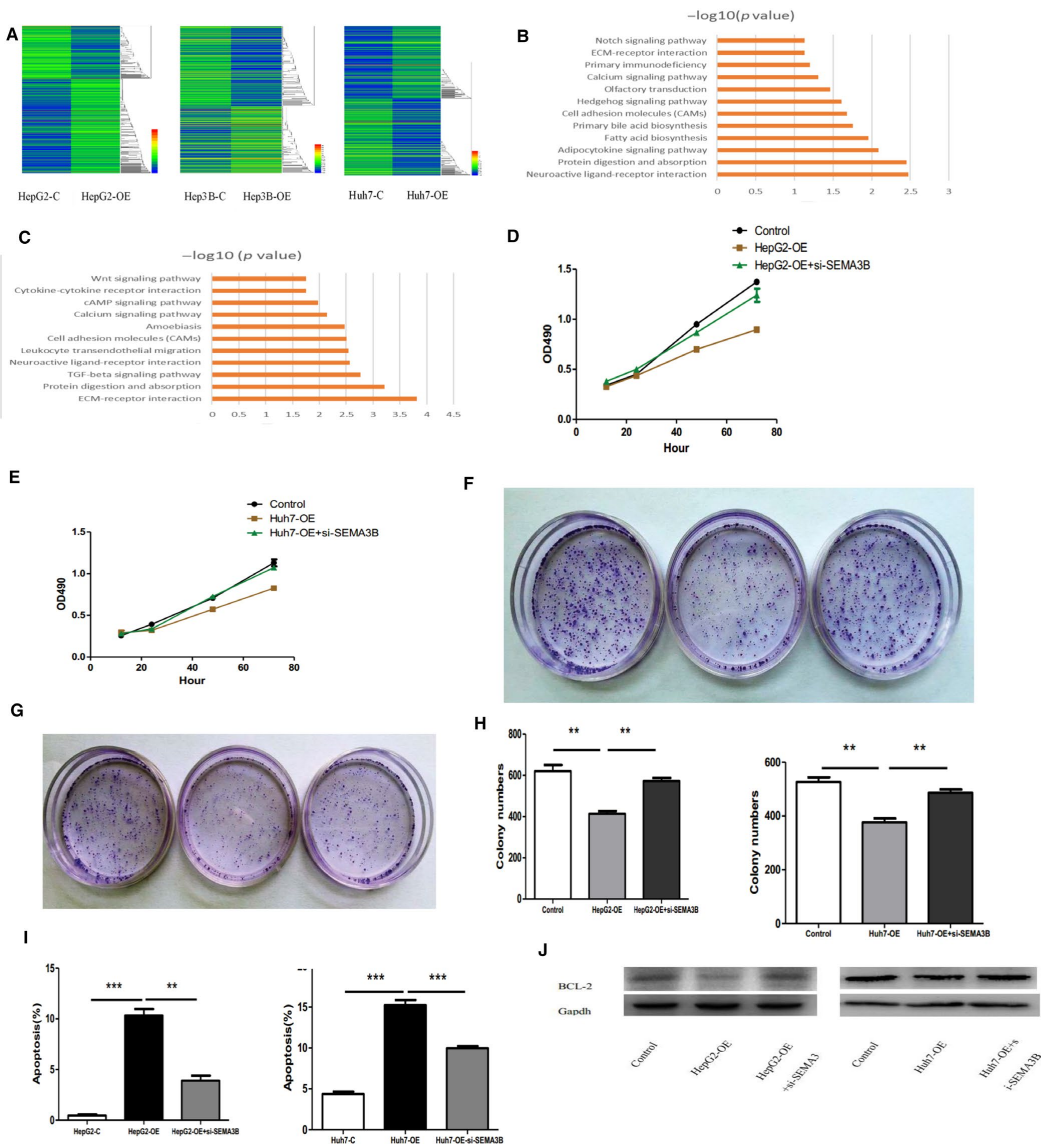
A, Heat maps of HepG2 and Huh7 cell expression profiles after EFEMP1 overexpression. B, EFEMP1‐related pathway analysis of HepG2 cells (partially ranked front pathway). C, EFEMP1‐related pathway analysis of Huh7 cells (partially ranked front pathway). D, Effect of EFEMP1 on HepG2 cell proliferation after transfection of si‐SEMA3B by MTT assay. E, Effect of EFEMP1 on proliferation of Huh7 cells after transfection of si‐SEMA3B by MTT assay. F, Clonal formation assay to detect the effect of EFEMP1 on HepG2 cell proliferation after transfection of si‐SEMA3B. G, Clonal formation assay to detect the effect of EFEMP1 on the proliferation of Huh7 cells after transfection of si‐SEMA3B. H, Inhibition of SEMA3B partially reversed the proapoptotic effect of EFEMP1. I, J, The propotion rate of apoptotic cells with the treament of si‐SEMA3B. K, The protein level of Bcl‐2 after transfection of si‐SEMA3B. *P < 0.05, **P  <  0.01, ***P  <  0.001

Through previous studies, we found that EFEMP1 was involved in the regulation of many biological processes in carcinoma,^21^, ^22^, ^23^ suggesting that EFEMP1 may play an important role in the development of HCC. When the transcription of EFEMP1 was inhibited, how did it cause phenotypic changes in HCC cells, and by what kind of signaling pathway to affect HCC? To solve this problem, we screened HepG2 cells and Huh7 cells overexpressing EFEMP1 for gene expression profiling. Differential gene screening was performed according to fold change >2, *P *≤* *0.05, and heat maps were drawn (Figure 6A), followed by GO analysis and pathway analysis of the differential genes. From the heat map results, when the expression of EFEMP1 changed, a lot of related downstream genes also changed. Pathway analysis showed that differential genes were enriched in neuroactive ligand receptor interaction, Axon guidance, and other signaling pathways (Figure 6B,C). The results suggested that these signaling pathways may be involved in the process by which EFEMP1 regulated the biological function of HCC cells. Among the members of these signaling pathways, we noticed the Semaphorin 3B (SEMA3B) gene, an important gene involved in cell death and aging. SEMA3B is a member of the Axon guidance pathway and the Neuroscience pathway. Many reports have reported that SEMA3B gene is involved in the regulation of cell senescence and apoptosis, and our previous cell experiments found the effect of EFEMP1 on the proliferation and apoptosis of HCC cells. We hypothesized that the regulation of EFEMP1 on the growth of HCC cells may require SEMA3B. SEMA3B inhibitory compound siRNA and control siRNA were transfected into EFEMP1 overexpressing cell lines, respectively. Subsequently, the growth of HCC cells was observed, and it was found that the growth rate of cells overexpressed EFEMP1 was slower, but the cells growth rate was increased after co‐transfected SEMA3B siRNA. MTT assay was used to detect the proliferation of HCC cells. The results showed that the inhibition of EFEMP1 on the proliferation of HCC cells was attenuated after inhibition of SEMA3B, suggesting that the inhibition of EFEMP1 on HCC cells may require the presence of SEMA3B (Figure 6D,E). Subsequently, the colony formation assay reflected that overexpression of EFEMP1 could inhibit the colony formation of liver cancer cell. However, inhibition of SEMA3B using siRNA could attenuate the effects of EFEMP1 (Figure 6F‐H).

Semaphorin 3B is considered to be a tumor suppressor gene that induces apoptosis in tumor cells. Our previous results found that EFEMP1 could promote the apoptosis of HCC cells, and EFEMP1 and SEMA3B synergistically regulated the proliferation and senescence of HCC cells. We hypothesized that EFEMP1 regulated apoptosis in HCC cells via SEMA3B. SEMA3B inhibitory compound siRNA and control siRNA were transfected into EFEMP1 overexpressing cell lines, respectively. The regulation of EFEMP1 on apoptosis of co‐transfected HCC cells was observed by Annexin‐V APC/7‐AAD double staining. The results showed that when the expression of SEMA3B was inhibited, the ability of EFEMP1 to induce apoptosis of HCC cells was weakened. Correspondingly, SEMA3B was able to counteract the effects of EFEMP1 overexpression on apoptosis‐related proteins. For example, overexpression of EFEMP1 reduced the expression of apoptosis inhibitory protein BCL‐2, but when SEMA3B was simultaneously inhibited, the expression level of BCL‐2 was restored (Figure 6I,J).

### 3.10 Inhibition of SEMA3B attenuated the effect of EFEMP1

**Figure 7:**
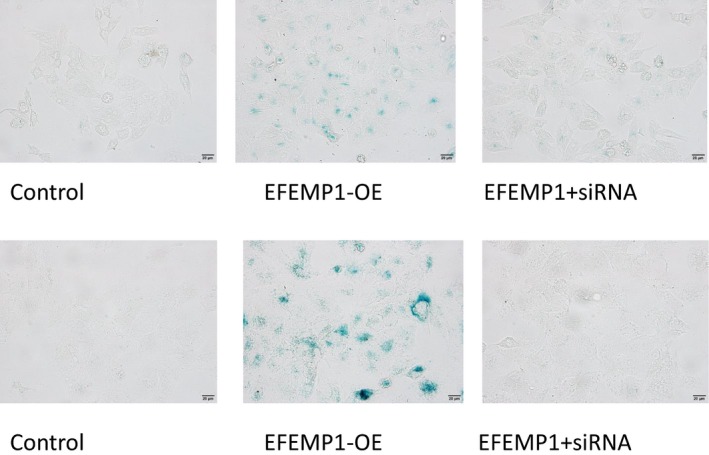
A, Cellular senescence β‐galactosidase staining assay. Blue staining of senescent cells showed that senescent cells increased after overexpression of EFEMP1, but senescent cells decreased after SEMA3B was inhibited (above row showed the results in HepG2, the below row showed the results in Huh7)

Semaphorin 3B is a gene associated with cell senescence. β‐galactosidase staining assay for cell senescence was performed to detect the effects of EFEMP1 and SEMA3B on HCC cells. When senescence appeared, cells would be stained blue. The results showed that when EFEMP1 was overexpressed, senescent cells were significantly increased. However, in HCC cells transfected with siRNA‐SEMA3B at the same time, the inhibition of the SEMA3B gene partially blocked the effect of EFEMP1 (Figure 7).

## 4 DISCUSSION

Hepatocellular carcinoma is a disease caused by a variety of causes, with complex pathogenesis, and there is currently no effective biomarker for the diagnosis of HCC and evaluation of HCC prognosis.

Epidermal growth factor‐containing fibulin‐like EFEMP1 has been found to be associated with a variety of tumors, and the changes in expression levels in different tumors are not the same. EFEMP1 is closely related to the clinicopathological features of various tumors. The initial experimental results showed that the mRNA level of EFEMP1 was decreased in HCC cells and primary HCC tissues. In our study, the sample size was expanded to carry out experiments, and it was found that both the mRAN and the protein expression level of EFEMP1 were decreased in primary HCC tissues. Combined with relevant clinical data, we found that the protein level of EFEMP1 may be related to TNM stage and Ki‐67 expression level in HCC patients. The expression of EFEMP1 was lower in patients with high stage, suggesting that EFEMP1 may play a protective role in the progression of HCC. Kaplan‐Meier survival analysis suggested that the protein level of EFEMP1 was positively correlated with the survival of HCC patients. Further multivariate Cox regression model analysis showed that EFEMP1 may be an independent prognostic factor for the outcome of HCC patients. In addition, the protein level of EFEMP1 in liver tissue was detected by tissue microarray. It was found that the staining intensity and positive rate of EFEMP1 gradually decreased with the severity of liver disease. When the liver became cancerous, its expression was significantly reduced. The results suggested that EFEMP1 may be involved in the process of liver disease. Therefore, EFEMP1 may become a potential predictor of prognosis, and it is expected to be more widely used in combination with clinically available predictors.

Abnormal methylation in the promoter region is an important cause of inactivation of some tumor suppressor genes. In 2010, it was reported in the literature that EFEMP1 was inhibited in HCC and DNA methylation may occur in the promoter region. Our experimental results showed that the EFEMP1 promoter region showed different degrees of methylation in HCC cells and primary HCC tissues. It is suggested that the inhibition of EFEMP1 transcription may be related to DNA methylation. However, the degree of methylation of EFEMP1 was different in different cells. EFEMP1 was methylated in HepG2, while DNA methylation was not observed in the promoter region of EFEMP1 in Huh7. After treatment of DNA methylation inhibitor, the expression of EFEMP1 in Huh7 cells was not restored. However, the expression of EFEMP1 in Huh7 was restored with the treatment of histone deacetylation inhibitor (data not shown). It is suggested that inhibition of EFEMP1 expression may be not only related to DNA methylation, but also may be related to histone deacetylation.^24^ Unlike gene mutations or deletions, the methylation status of the gene promoter region is reversible. Methyltransferase inhibitors not only restore the expression of silent genes, but also improve the drug resistance of some chemotherapeutic drugs. Therefore, our research may provide new ideas for the clinical treatment of malignant tumors.

Although the expression of EFEMP1 in HCC tissues was not associated with tumor size in the previous experiments, we found that the expression level of EFEMP1 was significantly negatively correlated with Ki‐67. Ki‐67 is an important marker of tumor cell proliferation. The effect of EFEMP1 on the proliferation of HCC cells was studied through cell experiments. The results showed that when the expression level of EFEMP1 was elevated, the proliferation of HCC cells was significantly inhibited. To further investigate the mechanism by which EFEMP1 regulates the proliferation of HCC cells, we found that EFEMP1 could affect apoptosis. There are usually two common pathways for apoptosis, one is the endogenous apoptotic pathway and the other is the exogenous apoptotic pathway.^25^ The classical exogenous pathway refers to the initiation of a death receptor localized on the cell surface after external stimulation of the cell. The death receptor is a member of the tumor necrosis factor receptor gene family, for example, Fas and TNFR1. In the death receptor‐mediated apoptotic pathway, Caspase‐8 is the key to initiation. When Caspase‐8 is activated, it can cause a Caspase protease cascade. The other is the mitochondrial apoptotic pathway. After cytochrome release, it affects the mitochondrial respiratory chain, reduces energy supply, and binds to apoptotic activating factor to form an apoptotic body, which causes the activation of Caspase‐9 and Caspase‐3. We found that overexpression of EFEMP1 increased the activation of Caspase‐9 and Caspase‐3, but the change of Caspase‐8 was not obvious, suggesting that EFEMP1 may regulate apoptosis through mitochondrial apoptosis pathway. The Bcl‐2 family members play a very important role in the process of mitochondrial apoptosis in cells.^26^ The Bcl‐2 family can be divided into two types, one is an anti‐apoptotic gene, such as Bcl‐2, Bcl‐xl, etc., and the other is a gene that promotes apoptosis, such as Bax, Bad, Bid, and the like. Our results showed that when the expression level of EFEMP1 was elevated, the expression of Bad, Bid, and Bax genes that promoted apoptosis was increased, while the expression of Bcl‐2 and Bcl‐xL genes inhibiting apoptosis was decreased. We found that the growth rate of HCC cells overexpressing EFEMP1 was slowed down in the subcutaneous tumor formation experiment in nude mice, suggesting that EFEMP1 may play a role in inhibiting cell proliferation in vitro and in vivo.

To further explore the downstream mechanism of EFEMP1 affecting the biological function of HCC cells, we used gene chip to detect changes in the expression profile of HCC cells after EFEMP1 overexpression. GO analysis and pathway analysis were performed based on differential genes. Some possible related signaling pathways have been discovered, such as Neuroactive ligand receptor interaction, Axon guidance, Wnt signaling pathway, and the like. Among the members of these signaling pathways, we noticed the SEMA3B gene. First, the microarray results suggested that SEMA3B was significantly altered in the chip, and it is included in the Axon guidance pathway and the Neuroscience pathway found by pathway analysis.^27^ Moreover, the SEMA3B gene was thought to play an important role in regulating cellular senescence and apoptosis.^28^ The role was considered to be a proapoptotic tumor suppressor gene. For example, SEMA3B was elevated in methotrexate‐induced cell senescence models.^29^ In lung cancer, wild‐type SEMA3B inhibited tumor growth by promoting apoptosis.^30^ The researchers also found that SEMA3B promoted apoptosis in breast cancer cells.^31^ Therefore, we considered that the regulation of EFEMP1 on HCC cells may be through SEMA3B based on the previous experiments. By interfering with the SEMA3B gene and inhibiting its expression, we found that SEMA3B could inhibit the inhibitory effect of EFEMP1 on the proliferation of HCC cells. Next, our results showed that the apoptosis of HCC cells induced by EFEMP1 required the presence of SEMA3B. In addition, the senescent cells were significantly increased by overexpression of EFEMP1, but inhibition of SEMA3B gene could block the effect of EFEMP1 in HCC cells. These results suggested that EFEMP1 may affect the proliferation and apoptosis of HCC cells through SEMA3B. However, it was unclear how EFEMP1 and SEMA3B interact with each other. Predictions by bioinformatics software, there are many common proteins that are related to EFEMP1 and SEMA3B (data not shown). We hypothesize that the regulation of EFEMP1 and SEMA3B may require the presence of a third party. These are worthy of our further research.

In conclusion, we found that the protein expression level of EFEMP1 in HCC tissues may be positively correlated with the survival of patients, and EFEMP1 may be an independent influencing factor for the prognosis of patients with primary HCC. EFEMP1 could inhibit the proliferation of HCC cells and promote the apoptosis of HCC cells. EFEMP1 regulates the apoptosis of HCC cells via the mitochondrial apoptotic pathway may be mediated by SEMA3B. Our study not only reveals the regulation of EFEMP1 on cell proliferation and apoptosis in HCC, but also provides a reliable theoretical basis for clinical diagnosis and targeted therapy of HCC.

## AUTHOR CONTRIBUTIONS

Jiangfeng Hu, Hengjun Gao, and Lungen Lu conceived and designed the experiments. Jiangfeng Hu, Bensong Duan, and Weiliang Jiang performed the experiments. Sengwang Fu and Hengjun Gao analyzed the data. Jiangfeng Hu and Lungen Lu wrote the paper. All authors read and approved the final manuscript.

## CONFLICT OF INTEREST

The authors declare that they have no competing interests.
